# NADPH Oxidase Activity in Cerebral Arterioles Is a Key Mediator of Cerebral Small Vessel Disease—Implications for Prevention

**DOI:** 10.3390/healthcare3020233

**Published:** 2015-04-15

**Authors:** Mark F. McCarty

**Affiliations:** Catalytic Longevity, 7831 Rush Rose Dr., Apt 316, Carlsbad, CA 92009, USA; E-Mail: markfmccarty@gmail.com; Tel.: +760-216-7272; Fax: +760-704-6379

**Keywords:** cerebral small vessel disease, NADPH oxidase, Nox2, eNOS, spirulina, potassium, arginine, cysteine, hypertension

## Abstract

Cerebral small vessel disease (SVD), a common feature of brain aging, is characterized by lacunar infarcts, microbleeds, leukoaraiosis, and a leaky blood-brain barrier. Functionally, it is associated with cognitive decline, dementia, depression, gait abnormalities, and increased risk for stroke. Cerebral arterioles in this syndrome tend to hypertrophy and lose their capacity for adaptive vasodilation. Rodent studies strongly suggest that activation of Nox2-dependent NADPH oxidase activity is a crucial driver of these structural and functional derangements of cerebral arterioles, in part owing to impairment of endothelial nitric oxide synthase (eNOS) activity. This oxidative stress may also contribute to the breakdown of the blood-brain barrier seen in SVD. Hypertension, aging, metabolic syndrome, smoking, hyperglycemia, and elevated homocysteine may promote activation of NADPH oxidase in cerebral arterioles. Inhibition of NADPH oxidase with phycocyanobilin from spirulina, as well as high-dose statin therapy, may have potential for prevention and control of SVD, and high-potassium diets merit study in this regard. Measures which support effective eNOS activity in other ways—exercise training, supplemental citrulline, certain dietary flavonoids (as in cocoa and green tea), and capsaicin, may also improve the function of cerebral arterioles. Asian epidemiology suggests that increased protein intakes may decrease risk for SVD; conceivably, arginine and/or cysteine—which boosts tissue glutathione synthesis, and can be administered as *N*-acetylcysteine—mediate this benefit. Ameliorating the risk factors for SVD—including hypertension, metabolic syndrome, hyperglycemia, smoking, and elevated homocysteine—also may help to prevent and control this syndrome, although few clinical trials have addressed this issue to date.

## 1. Functional Importance of Cerebral Small Vessel Disease in Brain Aging

Dysfunction and structural remodeling of cerebral arterioles seems likely to play a central role in the evolution of cerebral small vessel disease (SVD), characterized by silent lacunar infarcts, chronic or episodic ischemia, microbleeds, and the rarefaction of white matter giving rise to leukoaraiosis [1,2,3]. The progression of SVD has been linked to impaired cognitive function, sometimes evolving to vascular dementia, depression, as well as gait and balance disorders that increase risk for falling. Not surprisingly, the presence and progression of SVD is predictive of stroke risk.

The pathogenesis of SVD is distinct from that of large vessel atherogenesis, and is still somewhat mysterious. Elevation of LDL cholesterol, virtually a *sine qua non* for the development of atherosclerosis, has not emerged as a clear determinant of SVD risk, and SVD and vascular dementia have been relatively common in East Asian societies when coronary disease was rare. Systolic hypertension and aging are the best documented risk factors for SVD. Metabolic syndrome, smoking, and elevations of homocysteine and asymmetric dimethylarginine (ADMA) have also been associated with increased SVD risk in some studies.

## 2. Activation of NAPDH Oxidase in Cerebral Arterioles Is Central to the Pathogenesis of SVD

Although mitochondria generate superoxide at a steady low rate (which can be markedly amplified in certain pathological circumstances), the chief physiologically regulated sources of superoxide in most tissues are NADPH oxidase complexes, which occur in a range of isoforms. In cerebrovascular endothelium, the chief forms of NADPH oxidase that have been characterized are Nox1, Nox2, and Nox4-dependent; in humans (but not rodents), Nox5 is also expressed [4] Total NADPH oxidase activity in the cerebrovasculature, as opposed to systemic arteries, has been determined to be “profoundly greater” [5]. Studies with Nox2-knockout mice demonstrate that, though expression of Nox1 and Nox4 mRNAs in cerebrovascular endothelium has been found to be greater than that of Nox2, at least in basilar arteries, Nox2 activity can have important pathophysiological consequences [4,6].

Accumulating evidence cited below suggests that activation of the Nox2 form of NADPH oxidase in cerebral arterioles is a key mediator of the structural and functional derangements of cerebral arterioles associated with SVD. These arterioles, via adaptive vasodilation or constriction, play a crucial role in regulating brain blood flow in line with the metabolic demands of brain tissue. Excess superoxide production via NADPH oxidase impairs adaptive vasodilation by directly scavenging nitric oxide, and by inhibiting or uncoupling the endothelial NO synthase (eNOS). Oxidative stress, only in part because of its inhibitory impact on NO bioactivity, also appears to drive the structural remodeling (hypertrophy or inward remodeling) of cerebral arterioles observed in SVD, and can also promote leakiness of the blood-brain barrier.

Recent studies by Chan and Baumbach have been particularly illuminating in these respects. These researchers increased blood pressure selectively in the right carotid artery of mice by transverse aortic banding, and after 8 weeks observed the function and structure of cerebral arterioles on the surface of the right and left cortices—the left-sided arterioles being used as a control [7]. Some of the mice in this study were genetically Nox2 deficient, and some were pretreated with the eNOS inhibitor L-NAME. In wild-type mice, the vasodilatory response to acetylcholine was markedly blunted in the right arterioles as compared to those on the left, whereas response to the NO-generator sodium nitroprusside did not differ. Similarly, the cross-sectional areas of the right arteriolar vessel walls were increased relative to those of the left arterioles. An increase of oxidative stress (superoxide) was noted in the right-sided arterioles as compared to the left. Remarkably, these alterations of structure and function of the right-sided arterioles, as well as the associated increase in superoxide, were not seen in the Nox2-deficient mice, suggesting that they were dependent on evoked Nox2 activity. Pre-treatment with L-NAME decreased the vasodilatory response to acetylcholine in all arterioles, and Nox2 deficiency offered no protection in this regard—suggesting that the benefit of this deficiency reflected a preservation of NO bioactivity. Although L-NAME treatment caused a modest but statistically non-significant increase of vessel cross-sectional area in the left arterioles, Nox2 deficiency still prevented an increase of this area in the right-sided arterioles of L-NAME-treated mice—suggesting that oxidative stress contributed to vascular hypertrophy in the right-sided arterioles through mechanisms at least partially independent of NO activity. Studies employing rat aortic smooth muscle cells indicate that hydrogen peroxide, via sequential stimulation of Nox1 and ASK1 activity within these cells, can drive smooth muscle cell hypertrophy [8].

In aggregate, these findings suggest that hypertension activates Nox-2-dependent NADPH oxidase activity in cerebral arterioles, and the resultant oxidative stress blunts vasodilation mediated by eNOS-generated NO. This oxidative stress also induces arteriolar hypertrophy, an effect that is seen even in the absence of NO. How hypertension achieves this activation is not clear, but previous research has established that the cyclic strain associated with hypertension can activate NADPH oxidase in endothelial cells [9].

In an analogous study, these researchers showed that 4-week continuous infusion of angiotensin II led to increased oxidative stress and an inward remodeling—characterized by narrowing of the arterial lumen and decrease in external diameter—in the cerebral arterioles of mice; these changes were absent in Nox2-deficient mice [10]. A much earlier study had shown that topical application of angiotensin II impeded bradykinin-induced vasodilation in the pial arteries of rabbits, but that pre-treatment with the superoxide scavenger Tiron or the NADPH oxidase inhibitor DPI prevented this effect [11]. In an analogous study, angiotensin II infusion in mice blocked the homeostatically appropriate increase in cerebral blood flow evoked by a mechanical manipulation. No such effect was observed in mice pre-treated with losartan (AT1 receptor antagonist) or tiron, or in Nox2-deficient mice [12].

A number of other studies have shown that various pathogenic measures—type 1 diabetes, exposure to alcohol, nicotine, or cigarette smoke—inhibit the eNOS-dependent vasodilation evoked by acetylcholine or ADP in the pial arterioles of rats, but that administration of the NADPH oxidase inhibitor apocynin largely blunted this effect [13,14,15,16]. In mice fed a high-fat diet for up to 36 weeks to induce obesity and moderate hyperglycemia, acetylcholine-provoked vasodilation of cerebral arterioles was impaired; this effect was largely corrected by the superoxide scavenger tempol [17]. Arteriolar vasodilation remained normal when nox2-deficient mice were made similarly obese with a high-fat diet. In light of the fact that aging *per se* is a prominent risk factor for SVD, it is intriguing that eNOS-dependent vasodilation of cerebral arterioles is impaired in aged rats, as contrasted to adult rats; treatment with tempol, or the NADPH oxidase inhibitors apocynin and DPI, restored normal vasodilation in these aged rats [18]. An age-related up-regulation of NADPH oxidase activity in cerebral arterioles may therefore help to explain why SVD is most common in the elderly.

Hence, if the behavior of cortical cerebral arterioles in rodents is a reasonable model for the behavior of human cerebral arterioles, activation of Nox2-dependent NADPH oxidase activity in these arterioles appears likely to be a key mediator of the structural and functional arteriolar derangements that are at the root of SVD. Speaking in favor of this view are recent cross-sectional epidemiological studies demonstrating inverse correlations between serum bilirubin levels and risk for silent cerebral infarcts (primarily lacunar infarcts) and leukoaraiosis in humans [19,20]. Intracellularly, in low nanomolar concentrations, free bilirubin functions as an inhibitor of NADPH oxidase complexes [21,22,23,24]. The superior vascular health often correlated with elevated serum bilirubin is thought to reflect either a direct antioxidant effect of this bilirubin on cells, and/or an increased genetic propensity to generate bilirubin within cells via heme oxygenase activity [25,26,27,28].

## 3. What Factors Activate NADPH Oxidase in Cerebral Arterioles?

If this hypothesis has validity, it becomes important to define the range of pathogenic factors which can activate NADPH oxidase in cerebral arterioles. The impact of hypertension and angiotensin II in this regard has been discussed. It is notable that saturated fatty acids (mediators of the lipotoxicity of metabolic syndrome), homocysteine, semi-stable organic compounds in cigarette smoke, and hyperglycemia have been reported to boost NADPH oxidase activity in endothelial cells [29,30,31,32,33,34,35,36,37,38]. Potentially, such effects could rationalize epidemiology linking SVD to metabolic syndrome, diabetes, smoking, and elevated homocysteine. It has been postulated that marinobufagenin, an endogenously produced triterpenoid thought to mediate much of the pathogenicity of high-salt diets, can also increase endothelial NADPH oxidase activity [39,40,41]. Indeed, a short-term high-salt diet in Sprague-Dawley rats, which failed to raise systemic blood pressure, nonetheless inhibited the vasodilatory response of pial arterioles to acetylcholine or iloprost [42]. This study did not evaluate the role of NADPH oxidase in this effect. The high risk for stroke and vascular dementia in East Asian societies whose traditional diets are very high in salt and low in potassium is paralleled by a high prevalence of lesions in small intracerebral arteries noted in autopsy studies; risk for stroke in these societies appears to be higher than could be predicted from blood pressure per se [43,44,45,46].

A key feature of leukoaraiosis is increased blood–brain barrier permeability [47,48,49]. Hence, it is intriguing to note that endothelial oxidative stress tends to impede the formation of endothelial tight junctions [50,51,52,53,54]. Arguably, NADPH oxidase over-activity could be at the root of this phenomenon as well. Marinobufagenin has been reported to increase the permeability of brain endothelial cell monolayers [55].

## 4. Practical Strategies for Preventing Cerebral Small Vessel Disease

With respect to the prevention or control of SVD, it may be useful to consider what measures could be employed to suppress the NADPH oxidase activity of cerebral arterioles. Statin therapy in an adequately high dose has the potential to inhibit NADPH oxidase activity by suppressing the isoprenylation of Rac, which plays a role in the assembly of NADPH oxidase complexes [56,57]. A recent small open clinical study has concluded that treatment of patients with leukoaraisosis with 80 mg simvastatin daily can increase cerebral blood flow in both gray and white matter [58]—this despite the fact that LDL cholesterol is not a clear risk factor for SVD.

Cyanobacteria such as spirulina contain high concentrations of the biliverdin metabolite phycocyanobilin (PhyCB). Within cells, PhyCB is converted via biliverdin reductase to the close bilirubin homologue phycocyanorubin [59]. PhyCB has been shown to be a potent inhibitor of NADPH oxidase complexes in human cells, in a manner quite analogous to biliverdin/bilirubin [60,61]. Since there are no rich natural sources of biliverdin or bilirubin, and since these agents are expensive to synthesize, it is of particular interest that PhyCB can constitute about 0.6% of spirulina by dry weight (reflecting the fact that it functions to harvest light energy, much like chlorophyll does) [61]. Moreover, the remarkable range of anti-inflammatory and cytoprotective effects of orally administered spirulina (or of phycocyanin, the spirulina protein which contains PhyCB as a chromophore) reported in a number of rodent studies, encourage the view that orally administered PhyCB has the potential to act as a nutraceutical inhibitor of NADPH oxidase complexes [61,62].

In this regard, orally administered spirulina, and parenterally administered phycocyanin or PhyCB, has been shown to be markedly protective to the brain in rodent models of stroke, with or without reperfusion [63,64,65,66]. These findings can be rationalized by evidence that NAPDH oxidase activation is an important mediator of ischemia-reperfusion damage in stroke, and mediates the inflammation and oxidative stress in the stroke penumbra when the vascular occlusion is permanent [67,68,69]. Hence, it is reasonable to suspect that oral administration of whole spirulina, or spirulina extracts enriched in PhyCB, might be useful for preventing or controlling SVD. This hypothesis could be evaluated by noting the impact of spirulina/PhyCB on cerebral arteriolar structure and function in rodents subjected to hypertension, angiotensin II, or the other suspected risk factors for SVD cited above.

High potassium diets are associated with a notable decrease in stroke risk [70,71,72,73]. There is some evidence that a modest increase in serum potassium, such as that achievable with a high-potassium diet, can decrease endothelial NADPH oxidase activity while increasing eNOS activation, owing to a mild hyperpolarizing impact on the plasma membrane [74,75,76]. It would be of interest to determine the impact of potassium-rich diets in rodent models of cerebral arteriolar dysfunction. In spontaneously hypertensive rats fed a high-salt diet, an increase in potassium intake which failed to modify the elevated blood pressure of the rats nonetheless markedly lowered the number of brain infarcts and hemorrhages, reduced mortality, and decreased hypertrophy of mesenteric arterioles [70]. Intriguingly, the people of the Melanesian island of Kitava, who ingest very modest amounts of salt and have an exceptionally high potassium intake (from their dietary staple, yams), appear to be virtually free of hypertension, stroke, and dementia into advanced old age [77,78,79].

Arguably, measures which control hypertension, block angiotensin II activity, diminish elevated homocysteine, and ameliorate metabolic syndrome, may have potential for blocking NADPH oxidase activity in cerebral arterioles and preventing SVD; avoiding tobacco smoke and high salt intakes may also be useful in this regard. With respect to homocysteine, it is clear that controlling high-normal homocysteine with vitamin therapy (folate, B12, B6) fails to prevent coronary events; however, some but not all meta-analyses of controlled trials suggest that such supplementation may achieve a small reduction in stroke risk [80,81]. Hence, the possibility that homocysteine control could influence the course of cerebral SVD should not be dismissed out of hand. An association of the methylene tetrahydrofolate reductase polymorphism C677T with leukoaraiosis is of interest in this regard [82].

## 5. Further Measures for Supporting eNOS Activity

In addition, measures which support effective eNOS activity might be able to compensate to some degree for the adverse impact of oxidative stress on eNOS activity. Levels of serum asymmetric dimethylarginine (ADMA) are elevated in patients with cerebral SVD, compatible with the possibility that supplementation with citrulline (or arginine) could aid eNOS function in cerebral arterioles [83,84,85].

Although high-dose folate has utility for promoting recoupling of eNOS in vascular endothelium [86,87,88,89,90] (likely because the scavenging activity of reduced folates prevents peroxynitrite-mediated oxidation of tetrahydrobiopterin), it may not have access to the cerebral endothelium forming the blood–brain barrier. The high-capacity, low-affinity reduced folate carrier required for endothelial uptake of high concentrations of folate does not appear to be expressed by the lumenal membrane of cerebrovascular endothelium; rather, the high-affinity membrane-bound folate receptor, which is near-saturated at normal low-nanomolar plasma levels of folate, appears to mediate folate transport into cerebrovascular endothelium [91,92].

In rat studies, the episodic sheer stress associated with aerobic exercise training increases the expression of eNOS in both conductance and resistance arteries [93]. This may explain why such training enhances the vasodilatory responsiveness of cerebral arterioles in mice that are diabetic, exposed to nicotine, or subjected to transient focal ischemia of the brain [94,95,96]. The impact of exercise training on risk for cerebral SVD in humans appears to so far have received little attention from epidemiologists. However, one recent study found that daily time spent walking correlated inversely and dose-dependently with risk for stroke in elderly men [97].

Certain dietary flavonoids, such as cocoa flavanols (epicatechin) and quercetin, have the potential to stimulate eNOS activity by an interaction with the endothelial plasma membrane; [98,99,100] a report that ingestion of cocoa flavanols can acutely increase blood flow in certain brain regions suggests that this effect may be pertinent to cerebral arterioles [101]. Perhaps more importantly, most pertinent studies conclude that these agents—as well as the catechins in green tea—can amplify the endothelium-dependent vasodilation of systemic arteries triggered by shear stress or acetylcholine [98,102,103,104,105,106,107,108]. Of particular interest is a study demonstrating that in an atherosclerosis-prone strain of mouse, chronic catechin treatment improves flow-mediated and acetylcholine-mediated vasodilation of cerebral arteries, while improving the learning abilities of the mice [107].

Endothelial cells express transient receptor potential vanilloid 1 (TRPV1) receptors which allow influx of calcium when activated by heat, acidity, certain endogenous lipid metabolites, and the pungent phytochemical capsaicin [109]. *In vitro*, capsaicin induces increased expression and activation of eNOS in endothelial cells [110,111]. Moreover, in wild-type but not TRPV1 knockout mice, dietary capsaicin enhances endothelium-dependent vasodilation [112]. When fed at 0.02% of diet to spontaneously hypertensive stroke-prone rats, capsaicin increased the activation and expression of eNOS in cerebral arteries, decreased arteriolar hypertrophy, delayed stroke onset, and increased average survival [113]. These findings suggest that some adequate dietary intake of capsaicin, by promoting greater eNOS activity in vascular endothelium, could have potential for prevention of SVD.

## 6. The Impact of Dietary Protein

One convenient way to obtain more arginine for support of eNOS activity is simply to eat more protein. Protein can also provide cysteine, which, by increasing synthesis of the antioxidant glutathione synthesis in tissues, has the potential to promote ADMA catabolism and thereby disinhibit eNOS [114,115]. There is recent evidence that the efficiency of glutathione synthesis declines during aging, and that this effect can be compensated with supplemental cysteine [116]. Clinically, supplemental *N*-acetylcysteine (1.8 g daily) has been reported to potentiate the antihypertensive benefit of ACE inhibitors, and concurrent supplementation with *N*-acetylcysteine and arginine can decrease markers of endothelial activation and lower systolic blood pressure in hypertensive diabetics [117,118,119]. These considerations may help to rationalize the considerable evidence that diets high in protein tend to reduce risk for hypertension; plant-derived protein emerges as particularly protective in this regard, perhaps because it tends to be higher in cysteine and arginine [120,121,122].

It is reasonable to suspect that an increase of vascular glutathione content stemming from an increased dietary intake of cysteine, or from supplementation with *N*-acetylcysteine, could oppose some of the downstream pro-inflammatory effects of the superoxide generated by NADPH oxidase in small cerebral arteries. Hydrogen peroxide exerts such effects by oxidizing cysteine groups in signaling proteins, reversibly converting them to sulfenic acids; glutathione, and glutathione-dependent enzymes, act to revert these cysteine groups to their native form [123,124,125,126]. Hence, increased intakes of cysteine might complement measures which lessen NADPH oxidase activity in preventing SVD.

Indeed, the possibility that increased dietary protein intake—independent of its favorable impact on blood pressure—may reduce risk for cerebral SVD, merits consideration. In high-salt Asian societies whose traditional quasi-vegan diets tend to minimize their risk for atheroma in the coronary and cerebral arteries, cerebral SVD is quite common and is associated with a high incidence of lacunar infarct and hemorrhagic strokes [46]. Indeed, at any given level of blood pressure, this clinical picture has been far more common in these Asian societies than in Western nations [44,46,127]. High dietary salt intake and low potassium intake likely play a key role in this, but variables such as low LDL cholesterol and low intakes of animal fat and protein have been suggested as possible explanations. Perhaps it is low total protein intake, reflecting diets in which white rice predominates, that is the true culprit; Asian epidemiologists should devote further attention to this issue. In stroke-prone spontaneously hypertensive rats, thought to be a good model for lacunar stroke and hemorrhage associated with SVD, high dietary protein intakes postpone stroke and mortality [128,129]. Total protein has been linked to decreased stroke risk in some but not all prospective epidemiology; animal protein in particular emerges as protective in Asian epidemiology, as well as dietary correlates such as saturated fat and increased LDL cholesterol [130,131,132,133,134,135]. On the other hand, Western epidemiology associates red and processed meat consumption with increased stroke risk [136,137]. Conceivably, Asian epidemiology may be more illuminating than Western epidemiology with respect to SVD risk, since traditionally that is the chief cause of stroke and dementia in Asian quasi-vegan societies.

Protein could be protective owing to the effects of its constituent amino acids (such as arginine and cysteine), and/or perhaps because of its impact on systemic IGF-I levels [138]. IGF-I stimulates eNOS activity in endothelial cells via the PI3K-Akt pathway, prevents apoptosis in vascular smooth muscle cells, promotes elastin gene transcription, and can promote expression of the tight junction protein zona occludens-1 [139,140,141,142,143]—all effects which could be expected to have a favorable impact on SVD. However, epidemiologists have yet to explore the association of plasma IGF-I with risk for cerebral SVD *per se*. A Danish prospective study reported an elevated risk for ischemic stroke in subjects within the bottom quartile of IGF-I, and polymorphisms of the IGF-I gene have been associated with ischemic stroke risk in two studies [144,145,146]. It is pertinent to note that, when high-quality animal protein is added to a diet that otherwise is plant based, an increase in plasma IGF-I can be expected—whereas adding extra protein to a Western omnivore diet, already amply supplied with essential amino acids, will have minimal impact on IGF-I levels [147,148]. Perhaps that explains why dietary correlates of animal protein such as saturated fat and LDL cholesterol have been associated with decreased stroke risk in many Asian studies, but uncommonly in Western studies. In the Western, increased saturated fat and the elevated LDL cholesterol it gives rise to would be expected to promote atherosclerosis in large cerebral vessels, a more important determinant of stroke risk in the West.

Until further clarification is achieved, increased intakes of protein—from sources other than red or processed meats—can be recommended to those at risk for SVD. Indeed, there is recent evidence that increased protein intakes in the elderly may reduce their risk for frailty as well as total mortality [149]. On the other hand, the relatively low IGF-I levels associated with quasi-vegan diets of modest protein content seem likely to decrease total cancer risk, and may literally slow the rate of aging; such diets are also associated with low risk for atherosclerosis, diabetes, and possibly autoimmunity [148,149,150,151,152,153,154,155,156,157]. Perhaps a prudent overall strategy—as suggested by a recent epidemiological analysis from Fontana and colleagues—is to consume a quasi-vegan diet of modest protein content during young and middle adulthood, and then boost protein intake past age 70 [149]. Vegans would be well advised to keep their blood pressure under good control, as hypertension is the chief modifiable cause of cerebral SVD.

## 7. Conclusions

**Figure 1:**
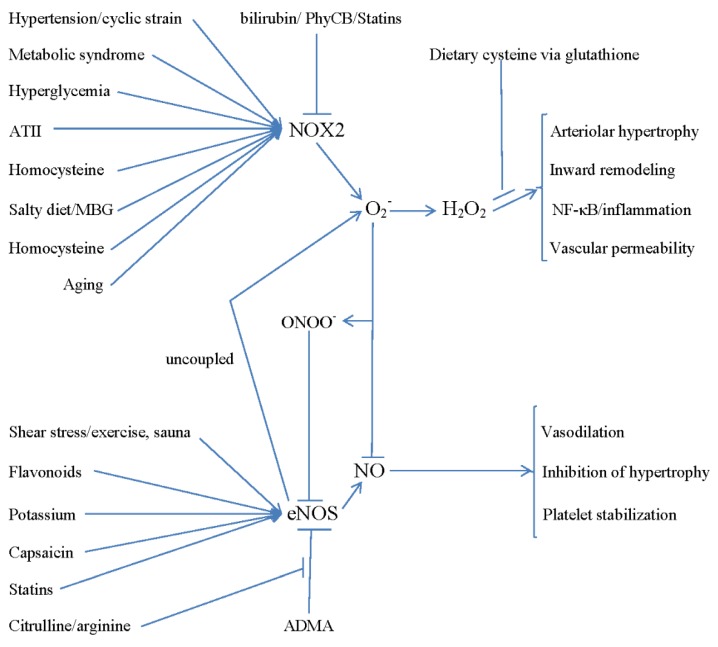
Mechanisms Regulating the Balance of Nox2 and eNOS Activity in Cerebrovascular Endothelium, and their Functional Consequences.

In summary, cerebral SVD, associated with lacunar infarcts, microbleeds, leukoaraiosis, and increased stroke risk, is a common cause of cognitive decline, dementia, depression, and gait abnormalities in the elderly. Structural and functional derangements of cerebral arterioles—impaired adaptive vasodilation, hypertrophy—appear to play a central role in this syndrome. Rodent studies strongly suggest that activation of Nox2-dependent NADPH oxidase activity in cerebral arterioles is a primary mediator of these derangements in SVD. This rodent research also suggests that hypertension, aging, angiotensin II, hyperglycemia, alcohol, and nicotine can promote NADPH oxidase activation in cerebral arterioles—in partial concordance with the current epidemiology addressing SVD. Oxidative stress generated by NADPH oxidase may also impair tight junction formation and thus promote the leakiness of the blood–brain barrier associated with SVD—possibly contributing to the impaired cognitive function characteristic of this syndrome. PhyCB from spirulina, as well as high-dose statin therapy, may have the potential to ameliorate the course of SVD by suppressing NADPH oxidase activity; control of hypertension may also be useful for this purpose, and the impact of high-potassium diets in this regard should be assessed. An increased intake of cysteine—from dietary protein or supplemental *N*-acetylcysteine—may boost glutathione synthesis and thereby alleviate the downstream pro-inflammatory effects of superoxide production, most notably in the elderly. Supporting eNOS activity of cerebral arterioles with supplemental citrulline, dietary flavonoids (cocoa epicatechin, quercetin, green tea), capsaicin, and exercise training, likely would have ancillary value in this regard. Evidently, measures which control mediating risk factors for SVD—the best documented of which is hypertension—may also prove useful for controlling this disorder. These interactions are summarized in Figure 1.
